# Recurrent cervical cancer treated with palliative chemotherapy: real-world outcome

**DOI:** 10.3332/ecancer.2020.1122

**Published:** 2020-10-13

**Authors:** Sharada Mailankody, Manikandan Dhanushkodi, Trivadi S Ganesan, Venkatraman Radhakrishnan, Vasanth Christopher, Selvaluxmy Ganesharajah, Tenali Gnana Sagar

**Affiliations:** 1 Department of Medical Oncology, Cancer Institute (WIA), Adyar, Chennai 600020, India; 2 Department of Medical Oncology, Kasturba Medical College, Manipal Academy of Higher Education, Manipal 576104, India; 3 Department of Radiation Oncology, Cancer Institute (WIA), Adyar, Chennai 600020, India; a http://orcid.org/0000-0003-2003-426X; b http://orcid.org/0000-0002-8192-3856

**Keywords:** chemotherapy, recurrent cervical cancer, palliative, outcomes, prognostic factors

## Abstract

**Introduction:**

Cervical cancer is the third most common cancer in India. There is limited data on the treatment of relapsed cervical cancer from India; therefore, we report the outcomes of patients with recurrent cervical cancer who were treated with palliative chemotherapy (CT).

**Materials and methods:**

This was a retrospective study of patients with recurrent cervical cancer who received palliative CT from January 2012 to December 2016. The demographic details, clinical profile and survival outcomes were collected. Patients were treated with carboplatin or paclitaxel and carboplatin. Local radiation was given for symptomatic patients. Patients were assessed for responses clinically and/or radiologically after three and six cycles of CT. Progression-free survival (PFS) and overall survival (OS) were calculated using the Kaplan–Meier method.

**Results:**

Forty-six patients with recurrent cervical cancer were included in this analysis, with a median follow-up of 9.4 months. The median age was 49.5 (25–65) years and the median disease-free interval was 31.3 (2–196) months. Biopsy confirmation of relapse was established in 63%. The median number of CT cycles was six. Twenty-four (52.2%) patients completed six cycles of CT. The overall response rate was 56.5%. Patients with a complete or a partial response were more likely to have PFS > 6 months (*p* < 0.0001). Median PFS and OS were, respectively, 8.4 (95% CI 6.1–10.7) months and 10.3 (95% CI 6.8–13.8) months. The completion of all cycles of CT and the site of metastasis (nodal vs. visceral or combined) were found to be associated with OS.

**Conclusion:**

Palliative CT with paclitaxel carboplatin is a safe and effective option in Indian patients with recurrent cervical cancer, with more than half of the patients completing the prescribed CT. Further prospective trials may be required to place this treatment in the right context, in this era of immunotherapy and targeted therapy. However, knowing the outcomes in our population and prognostic factors will help in better prognostication of patients, thereby channelling our limited resources where necessary.

## Introduction

Cervical cancer continues to be a significant cause of morbidity and mortality in India, despite the introduction of screening strategies in the community [1, 2]. It is the third most common cancer in India. There are 10,000 new cases registered per year, and it also has a higher mortality rate in India [3, 4]. It is second only to breast cancer among the causes of cancer mortality among females in 12 of the Indian states [1].

In India, the epidemiology of the disease has a distinct pattern, with more advanced stages at presentation and a definite rural predominance [1, 5, 6]. Also, the availability and organisation of oncological care are different in lower and middle-income countries (LMICs) compared to high-income countries, especially when it comes to medical oncology services [7]. Hence, the management of recurrent cervical cancer poses unique challenges in India and merits exploration.

The treatment of recurrent cervical cancer may require multimodality care, with chemotherapy (CT)/immunotherapy, radiotherapy (RT) or surgery; the exact sequence of which has not been established as yet [8, 9]. The prognosis of recurrent cervical cancer depends on multiple factors like performance status (PS), prior cisplatin use, pelvic disease, race, sites of metastasis and disease-free interval (DFI) [10–13]. With the approval of immunotherapy for recurrent cervical cancer, systemic treatment options now include platinum-based doublet CT, bevacizumab and immunotherapy [8]. Also, in resource-limited settings, the use of oral metronomic chemotherapy (OMC) has been described [14]. The overall response rates (ORR) from 15% to 65% have been described for various protocols in various settings of care [15–24]. There is paucity of data regarding the outcomes of systemic treatment of recurrent cervical cancer from India [20]. It is important to know the prognostic factors and outcomes of treatment so that our limited resources can be channelled towards treating the right patients. We undertook this analysis to explore the outcomes of CT in patients presenting with recurrent cervical cancer.

## Materials and methods

We collected data from the individual case records of patients with recurrent cervical cancer who were treated with palliative CT from January 2012 to December 2016. The demographic details, clinical profile and the treatment details were collected. The recurrence was confirmed with a fine needle aspiration or biopsy for those with late recurrences in accessible sites. The restaging investigation was by either contrast-enhanced computed tomography or positron emission tomography/computed tomography.

### Treatment protocol

After a multidisciplinary tumour board (MDT) discussion, patients were treated with palliative CT, with either single-agent carboplatin or paclitaxel and carboplatin (PC). The dose was carboplatin (area under the curve (AUC) 5) or paclitaxel (175 mg/m^2^) and carboplatin (AUC 5) every 3 weeks for six cycles depending on age, comorbid illness and PS. Patients were assessed for responses clinically and radiologically after three and six cycles of CT. Palliative radiation was given when indicated.

### Statistical analysis

Progression-free survival (PFS) was calculated from the date of starting CT until the date of last follow-up, progression or death. Overall survival (OS) was calculated from the date of starting CT until the date of last follow-up or death due to any cause. Follow-up was censored in August 2017. Actuarial survival was computed using the Kaplan–Meier method; prognostic factors were compared using the log-rank test. Multivariate analysis was carried out using the Cox proportional hazards regression model. All statistical analyses were carried out using SPSS version 13 (IBM Inc.), and a *p*-value < 0.05 was considered to be significant.

## Results

### Baseline characteristics

A total of 46 patients with recurrent cervical cancer were included in this analysis (Table 1).

Median age at presentation was 49.5 years (range: 25–65 years). The stages at presentation were stage I: 5 patients (10.9%); stage II: 32 patients (69.6%) and stage III: 9 patients (19.6%). All patients were treated with pelvic external beam radiotherapy with 45–50 Gy, followed by three high-dose-rate intracavitary applications done to deliver 21 or 24 Gy at point A. Target Eqd2 aimed was 80 Gy. Concurrent CT with weekly cisplatin was given to 37 patients (80.4%). Parametrial radiotherapy was given to 29 (63%) patients and paraaortic RT to 7 (15.2%) patients.

Median DFI was 31.3 months (range: 2–196 months). The DFI was less than a year in 19.6% (*n* = 9) and more than a year in 80.4% (*n* = 37). Asymptomatic and symptomatic relapses were 67.4% (*n* = 31) and 32.6% (*n* = 15), respectively. Single site of relapse was seen in 16 (34.8%) patients. The sites of relapse were lung in 27 (58.7%) patients, distant nodes in 24 (52.2%) patients, paraaortic nodes in 21 (45.7%) patients, local in 5 (10.9%) patients, bone in 5 (8.7%) patients and liver/adrenal in 3 (6.5%) patients. Of the patients with node-only metastasis (*n* = 13, 28.3%), five (10.9%) patients had paraaortic and supraclavicular metastasis, five (10.9%) patients had isolated paraaortic and one (2.2%) patient each had supraclavicular node, mediastinal node and inguinal node relapse. Tissue confirmation of recurrence was available in 29 (63%) patients.

The CT delivered was PC to 40 (87%) patients and single-agent carboplatin to 6 (13%) patients. Four (8.7%) patients were started initially on single-agent carboplatin, and then escalated to PC from the second cycle. Bevacizumab was given to one patient. The median number of treatment cycles was 6 (range: 1–6 cycles) and 24 (52.2%) patients completed all six cycles. Palliative radiation was given to 20 (43.5%) patients; except two patients with isolated local recurrence who were given pelvic/local RT, the other patients (*n* = 18) were given RT to the site of relapse.

### Response rates and survival outcomes

**Figure 1.**
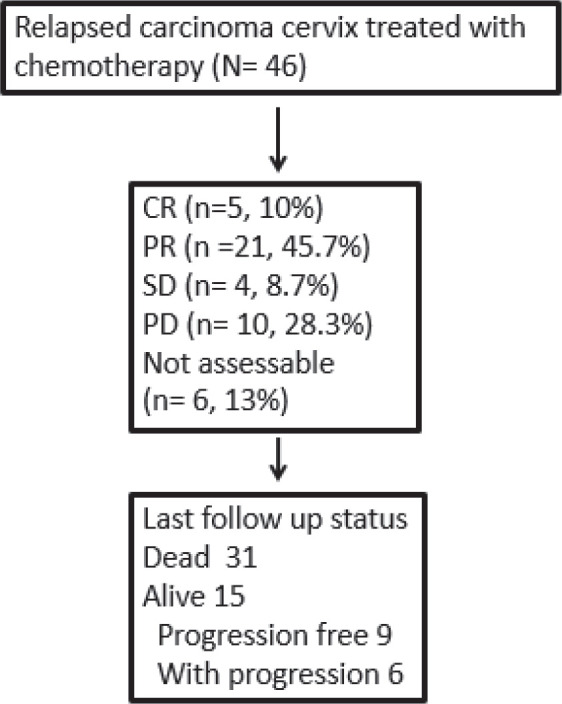
Responses and outcomes of patients with relapsed cervical cancer.

**Figure 2.**
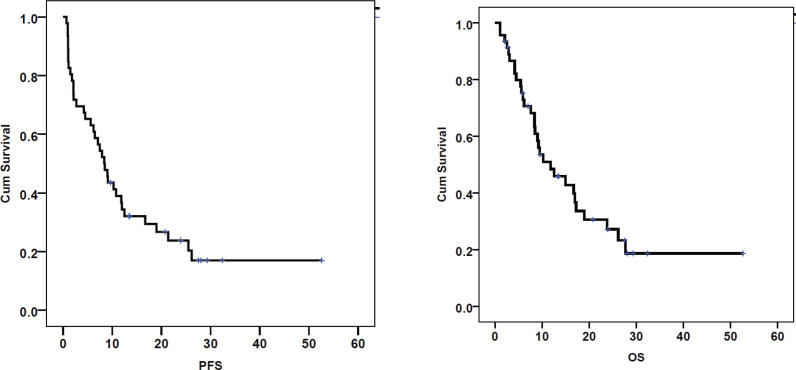
PFS and OS calculated using the Kaplan–Meier curves in patients with recurrent cervical cancer treated with palliative chemotherapy (*n* = 46).

The ORR was 56.5%, with a complete response in 5 (10.9%) patients (Figure 1). Patients with response (CR/PR) to CT were more likely to have a survival of >6 months (*p* < 0.00001). At the end of the study period, 15 (32.6%) patients were alive. The median follow-up was 9.4 months (range: 1.1–52.6). The median PFS and OS rates were 8.4 (95% CI 6.1–10.7) months and 10.3 (95% CI 6.8–13.8) months, respectively (Figure 2). The rates of one-year PFS and OS were 34.3% and 48.4%, respectively.

### Factors affecting OS

**Figure 3.**
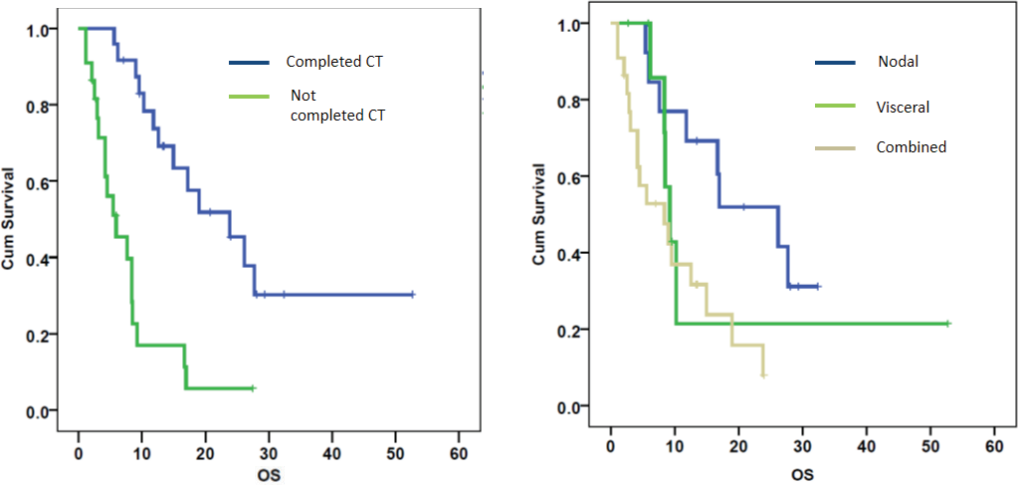
OS calculated using the Kaplan–Meier curves for patients based on a) completion of all cycles of chemotherapy (CT) (*p* = 0.000) and b) site of metastasis (*p* = 0.001).

On univariate analysis, the response to CT, site of metastasis and the completion of CT correlated with OS (Table 2). The patients with node-only metastasis were found to have a better OS than patients with visceral metastases or combined visceral–nodal metastasis. On multivariate analysis, the factors affecting survival were completion of CT and metastasis sites (Figure 3). Age, stage, tumour histology, grade, DFI and addition of RT did not correlate with survival.

### Toxicity details

Twenty-one (45.7%) patients had CT-related side effects, with 14 (30.4%) patients having neuropathy. Grade 3/4 side effects were seen in six (13%) patients, two (4.3%) patients with neuropathy, two (4.3%) patients with myelosuppression and two (4.3%) patients with metabolic complications. There was one death due to sepsis; however, this was in a non-neutropenic setting.

## Discussion

This study reports the outcomes of CT in recurrent cervical cancer from a tertiary cancer care hospital in India. With CT, there was an ORR of 56.5%, with CR in 10.9% patients. The median PFS and OS were 8.4 months and 10.3 months, respectively. Half of the patients (52.2%) were able to complete six cycles of CT. We also identified prognostic factors associated with survival.

A study in Japan found an RR of 70% in a trial using PC for recurrent cervical cancer, with a median PFS of 7 months [16]. In another Japanese trial, comparing PC with paclitaxel/cisplatin, an RR of 62.6% with median PFS and OS of 6.2 months and 17.5 months, respectively, was reported [17]. However, while the current study had 80% of the patients exposed to cisplatin, the above-mentioned trial included only 48% of such patients. In a systematic review comparing cisplatin and carboplatin-based therapies, an RR of 48.5% and mean PFS and OS of 5 months and 10 months, respectively, was seen for carboplatin-based therapy [18]. In another Indian study analysing the outcomes of both pre-treated and de-novo metastatic cervical cancer with PC, an RR of 26.7% with a median PFS of 6 months and OS of 11 months was reported [20].

The RR in our study was higher than that reported by the GOG trial which used cisplatin-based doublets and other retrospective studies [20, 21, 25]. However, our analysis did not include patients with persistent disease after the completion of chemoradiation therapy. Moreover, only around 20% of the patients in our study had a DFI of <6 months.

Although nearly half of the patients developed toxicities, there was only one toxic death. While neuropathy was the most significant toxicity in our study (30% of the patients), only two patients had grade 3 or 4 neuropathy. Although a Brazilian study has also reported 30% neuropathy with the use of PC, this percentage is higher than that reported by few other studies [17, 24]. Prior cisplatin treatment in 80% of our patients may account for the higher rates of neuropathy in our patients.

Interestingly, with the use of single-agent OMC (etoposide or cyclophosphamide) in a similar population, an RR of 23% and a median OS of 14.5 months was reported [14]. Since our study used two active agents, the RR may have been higher. The survival time seems similar across these studies.

In the current study, patients with node-only metastasis had a significantly better survival compared to those with visceral metastasis. Also, there was an improved survival in those who completed chemotherapy. Not surprisingly, the patients with CR/PR also had longer survival. These factors were reported in prior studies also [11–13]. However, age, histology, grade, DFI and prior cisplatin use were not found to be prognostic in the current study, which is contrary to the earlier studies [10].

This study represents the real-world outcome of recurrent cervical cancer treated with palliative CT. Cervical cancer in India is rurally predominant; hence, expensive treatment options, like immunotherapy and bevacizumab, may not be cost-effective, although low-cost biosimilar bevacizumab molecules are now available. Many patients have to travel long distances to reach oncology centres, thus developing and optimising simple yet effective protocols are very important for treatment compliance [7]. Moreover, the out-of-pocket expenditures are high for the treatment of cancer in India; although the cost of CT may be subsidised or free, the treatment process may cripple the family financially [25]. Hence, knowing the outcomes in our country and prognostic factors may help us to choose the appropriate patients for the initiation of palliative CT.

Further data are needed regarding the outcomes and ideal protocols of OMC with pharmacokinetics in this population, as we have in head and neck cancer patients [26]. However, till such data become available, the use of well-tolerated simple CT protocols may form the cornerstone for effective palliation of such patients.

The limitations of this study are retrospective design, small sample size and limited use of targeted therapy.

## Conclusion

PC is a useful treatment option in patients with recurrent cervical cancer. Lymph nodal metastasis and completion of therapy were associated with better outcomes. Further prospective studies are needed in India and other LMICs to exactly define the role of CT and the selection of the right patients for initiation of treatment. Identification of outcomes and prognostic factors in our population will help us in the optimal utilisation of available resources.

## List of abbreviations

LMICs: Lower- and middle-income countries
DFI: Disease-free interval
OMC: Oral metronomic chemotherapy
AUC: Area under the curve
PS: Performance status
CR: Complete response
PR: Partial response
PFS: Progression-free survival
OS: Overall survival
ORR: Overall response rate
PC: Paclitaxel carboplatin
CT: Chemotherapy

## Conflicts of interest

None of the authors have any conflict of interest.

## Funding

None.

## Figures and Tables

**Table 1.** Baseline characteristics of patients with recurrent cervical cancer.

Characteristic	No of patients (%) (*n* = 46)
Age (median(range))	49.5 (25–65)
Stage*	
I	5 (10.9)
II	32 (69.6)
III	9 (19.6)
Grade *	
II	5 (10.9)
III	41 (89.1)
Histology*	
SCC	37 (80.4)
PDC	4 (8.7)
Adenocarcinoma	5 (10.9)
Cisplatin*	37 (80.4)
Parametrial RT*	29 (63)
Paraaortic RT*	7 (15.2)
Mean ICAs*	2.6

* Data presented as *n* (%)

**Table 2.** Results of univariate and multivariate analysis of factors affecting survival.

Factor	OS (95% CI)	*p* value (for univariate analysis)	*p* value (for multivariate analysis)
*Completion of CT*			
Yes (*n* = 24)	23.8 (13.6–34.1)	0.000	0.000
No (*n* = 22)	5.9 (1.7–10.1)		
Site of metastasis*			
Nodal (*n* = 13)	26.2 (11.8–40.5)	0.034	0.001
Visceral (*n* = 9)	9.2 (7.3–11.1)		
Combined (*n* = 22)	8.4 (2.0–14.8)		
Response to therapy**			
SD/PD (*n* = 15)	5.9 (2.4–9.4)	0.028	0.843
CR/PR (*n* = 26)	17.2 (11.8–22.6)		
*DFI*			
Less than 1 year (*n* = 9)	8.4 (0.0–18.6)	0.106	NA
More than 1 year (*n* = 37)	14.9 (6.7–23.2)		
*Age*			
Less than 50 years (*n* = 21)	14.9 (6.4–23.5)	0.305	NA
More than 50 years (*n* = 25)	9.0 (3.8–14.2)		
*Symptomatic relapse*			
Yes (*n* = 15)	9.2 (6.1–12.4)	0.191	NA
No (*n* = 31)	12.6 (2.6–22.6)		
*Histology*			
Squamous (*n* = 37)	11.8 (5–18.6)	0.598	NA
Non SCC (*n* = 9)	17.2 (7.6–26.8)		
*Cisplatin upfront*			
Yes (*n* = 37)	9.5 (4.8–14.2)	0.628	NA
No (*n* = 9)	14.9 (5.7–24.2)		

* Excluding the two patients with isolated local recurrence

** Excluding the five patients whose response could not be assessed

**Table 3.** Toxicity details in patients given palliative chemotherapy.

Toxicity	No of patients (%)
Neuropathy	
Grade 1/2	12 (26)
Grade 3/4	2 (4)
Haematological	
Grade 3/4 myelosupression	2 (4)
Grade 2 thrombosis	1 (2)
Grade 3 colonic perforation	1 (2)
Grade 2 renal dysfunction	1 (2)
Grade 3 dyselectrolytemia	1 (2)
Death	1 (2)
